# Exosomal PD-L1 in cancer and other fields: recent advances and perspectives

**DOI:** 10.3389/fimmu.2024.1395332

**Published:** 2024-04-25

**Authors:** Man-Man Lu, Yu Yang

**Affiliations:** ^1^ Center for Reproductive Medicine, The First Affiliated Hospital of Zhengzhou University, Zhengzhou, Henan, China; ^2^ Department of Hepatobiliary and Pancreatic Surgery, The First Affiliated Hospital of Zhengzhou University, Zhengzhou, Henan, China

**Keywords:** exosome, PD-L1, immune checkpoint, exosomal PD-L1, immunotherapy, resistance, biomarker

## Abstract

PD-1/PD-L1 signaling is a key factor of local immunosuppression in the tumor microenvironment. Immune checkpoint inhibitors targeting PD-1/PD-L1 signaling have achieved tremendous success in clinic. However, several types of cancer are particularly refractory to the anti–PD-1/PD-L1 treatment. Recently, a series of studies reported that IFN-γ can stimulate cancer cells to release exosomal PD-L1 (exoPD-L1), which possesses the ability to suppress anticancer immune responses and is associated with anti-PD-1 response. In this review, we introduce the PD-1/PD-L1 signaling, including the so-called ‘reverse signaling’. Furthermore, we summarize the immune treatments of cancers and pay more attention to immune checkpoint inhibitors targeting PD-1/PD-L1 signaling. Additionally, we review the action mechanisms and regulation of exoPD-L1. We also introduce the function of exoPD-L1 as biomarkers. Finally, we review the methods for analyzing and quantifying exoPD-L1, the therapeutic strategies targeting exoPD-L1 to enhance immunotherapy and the roles of exoPD-L1 beyond cancer. This comprehensive review delves into recent advances of exoPD-L1 and all these findings suggest that exoPD-L1 plays an important role in both cancer and other fields.

## 1 Introduction

Programmed cell death protein 1 (PD-1) is one of the most famous co-inhibitory receptors on the surface of activated T cells (1). Ligands of PD-1 include PD-L1 and PD-L2. Both cancer cells and cancer stroma express PD-L1 and PD-L2. However, antibodies against PD-1, which block both PD-L1 and PD-L2, show no obvious clinical advantage than antibodies against PD-L1, indicating PD-L1 is the dominant ligand of PD-1 in human tumor microenvironment (2). Under physiological condition, the PD-1/PD-L1 signaling is involved in immune homeostasis and plays important role in immune tolerance. Upon recognition of antigens, T cells rapidly express PD-1 to modulate the strength of T cell activation (TCA). Cancer cells express tumor-related antigens continuously and, in such a setting of chronic antigen encounter, T cells express high level of PD-1 and become inept progressively. Accordingly, T cells lose effector functions and develop into an exhausted state (3). A seminal study first reported cancer cells can release exosomes that carry PD-L1 on their surface (4). This kind of exosomal PD-L1 (exoPD-L1) can be stimulated by interferon-γ (IFN-γ) and the amount of circulating exoPD-L1 correlates with level of IFN-γ positively. ExoPD-L1 can systemically suppress the functions of T cells, leading to cancer growth (4). Later on, a series of studies confirmed the ability of exoPD-L1 to promote cancer growth in breast cancer, gastric cancer (GC), non-small cell lung cancer (NSCLC), etc. (5–7). Currently, blocking PD-1/PD-L1 interaction by monoclonal antibodies is applied to cancer treatment. Despite remarkable curative effect, some patients are non-responders. By recapitulating the effect of cell surface PD-L1, exoPD-L1 is involved in lowering the treatment response.

In this review, we summarize the PD-1/PD-L1 signaling and the immune treatments of cancers, especially immune checkpoint inhibitors (ICIs) targeting PD-1/PD-L1 signaling. Furthermore, we review the action mechanisms and regulation of exoPD-L1. Additionally, we discuss the role of exoPD-L1 as biomarkers for diagnosis, prognosis and predicting responses of immunotherapies. Finally, we review the methods for analyzing and quantifying exoPD-L1, therapeutic strategies targeting exoPD-L1 to enhance immunotherapy and the roles of exoPD-L1 beyond cancer. This comprehensive review delves into recent advances of exoPD-L1 and all these findings suggest that exoPD-L1 plays an important role in both cancer and other fields.

## 2 PD-L1/PD-1 signaling

Both PD-1 and PD-L1 are type I transmembrane proteins and are categorized into the immunoglobulin (Ig) superfamily. PD-1 is composed of an Ig-V like extracellular domain, a transmembrane domain and a cytoplasmic domain (1, 8). PD-L1 consists of an Ig-V and Ig-C-like extracellular domain, a transmembrane domain and a short cytoplasmic tail. The cytoplasmic domain of PD-1 harbors two tyrosine-based signaling motifs whereas the short cytoplasmic tail of PD-L1 possesses no canonical signaling motifs (1, 8).

**Figure 1:**
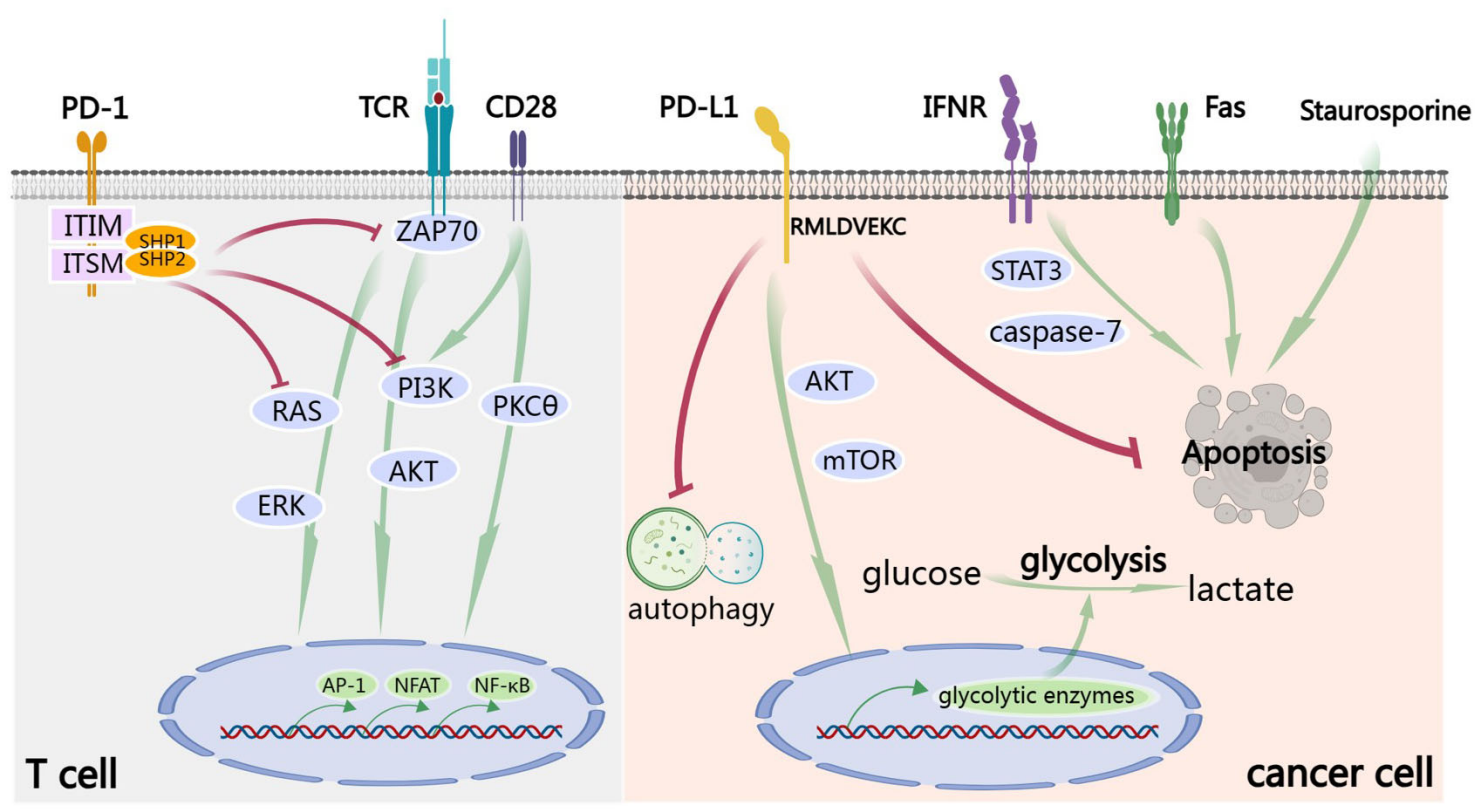
PD-1/PD-L1 signaling. In T cell, TCA requires the signals from TCR and CD28. After the engagement by PD-L1, ITIM and ITSM of the cytoplasmic domain of PD-1 are phosphorylated. Subsequently, the SHP-1 and SHP-2 protein tyrosine phosphatases are recruited to dephosphorylate activation signals from TCR and CD28, including ZAP70 and to inhibit downstream PI3K/AKT, RAS/ERK and PKCθ signaling. Finally, all these result in decreased activation of transcription factors, including AP-1, NFAT and NF-κB, which are involved in activation, proliferation, effector functions and survival of T cell. Signaling down from PD-L1 is called ‘reverse signaling’. Take cancer cell for example. PD-L1 signaling activates AKT/mTOR signaling and promotes expression of glycolytic enzymes and subsequent glycolysis. Furthermore, PD-L1 signaling promotes cancer by inhibiting autophagy and apoptosis, including IFN-induced apoptosis by STAT3/caspase-7-dependent pathway, Fas-induced and Staurosporine-induced apoptosis. RMLDVEKC motif of intracytoplasmic domain of PD-L1 is of great importance in inhibition of apoptosis.

TCA requires two signals. The first signal is the peptide-major histocompatibility complex class I (MHC I) presented on the surface of an antigen-presenting cell (APC) interacts with the T cell receptor (TCR). The second signal requires immune co-stimulating molecules such as CD80 and/or CD86 to interact with CD28 on T cell. The cytoplasmic domain of PD-1 possesses two independent phosphorylation sites: the immunoreceptor tyrosine-based inhibitory motif (ITIM) and the immunoreceptors tyrosine-based switch motif (ITSM) (8). After the engagement of PD-1 by PD-L1, conformational change in PD-1 is induced, leading to phosphorylation of ITIM and ITSM (9). Subsequently, the Src homology 2 domain-containing protein tyrosine phosphatase 1 (SHP-1) and SHP-2 are recruited by the phosphorylated tyrosine motifs to attenuate T cell-activating signals (2). Specifically, the recruited SHP-1 and SHP-2 dephosphorylate activation signals that occur through the TCR and CD28, such as ZAP70, inhibiting downstream phosphoinositide 3-kinase (PI3K)/AKT signaling (10, 11), RAS/extracellular-signal-regulated kinase (ERK) signaling and PKCθ signaling (12, 13). Collectively, this results in decreased activation of transcription factors, including AP-1, NFAT and NF-κB, which are involved in activation, proliferation, effector functions and survival of T cell (**Figure 1**). Importantly, CD28 was reported to be a primary target for dephosphorylation by the SHP-2 phosphatase (14) and anti-PD-L1 therapy rescued exhausted T cells by CD28 signaling (15). It should be mentioned that CD80 is also a receptor of PD-L1 and can deliver inhibitory signals to activated T cells (16, 17).

As a matter of fact, after binding of PD-L1 to PD-1, a so-called ‘reverse signaling’ through PD-L1 exists but has been less studied because of the absence of canonical signaling motifs in the short cytoplasmic tail of PD-L1. There are some excellent studies exploring this area. Recombinant PD-1 delivers pro-survival signals to cancer cells through PD-L1 and endows cancer cells with resistance against T cell-mediated killing (18). Furthermore, cancer cells stimulated by PD-L1 are also refractory to apoptosis mediated by Fas ligation and the protein kinase inhibitor Staurosporine (18). Enhancing glycolysis in cancer leads to glucose consumption and is sufficient to metabolically restricts functions of T cells, allowing cancer progression (19). PD-L1 signaling is closely associated with glycolysis in cancer cells and maintains AKT/mTOR signaling, supporting the translation of glycolytic enzymes (19). Blocking PD-L1 by antibody reduces mTOR activity and glycolytic metabolism in cancer cells (19). PD-L1 can also promote cell proliferation and survival by inhibiting autophagy and activating mTOR in the absence of PD-1 (20). IFN exerts cytotoxicity to cancer cells through a STAT3/caspase-7-dependent pathway, which can be interfered with PD-L1 signaling via RMLDVEKC motif of intracytoplasmic domain (21) (**Figure 1**).

## 3 Immune checkpoint inhibitors targeting PD-1/PD-L1 signaling

In the past few decades, immune treatments of cancers have achieved tremendous success in clinic (22, 23). As early as 1891, William B Coley, an orthopedic surgeon, injected bacteria into tumors to treat cancer, marking the beginning of immunotherapy of cancer (24). 1994, Allison’s team revealed the ground-breaking discovery that blocking CTLA-4, an immune checkpoint, promoted the antitumor activity of T cells and inhibited tumor growth. Accordingly, this approach was referred to as ‘immune checkpoint blockade’ (ICB) by Allison for the first time (23). In 2011, the first ICI targeting CTLA-4, ipilimumab, was approved for melanoma treatment. As the discovery of the PD-1/PD-L1 signaling pathway, ICIs targeting PD-1/PD-L1 became the most widely applied immunotherapy, with 6 drugs approved in the United States and 4 PD-1 inhibitors in China (23). In addition to ICIs, chimeric antigen receptor T-cell (CAR-T) therapy represents another rigorously evaluated immunotherapy strategy and achieve extraordinary clinical responses in hematological cancers (25). In brief, chimeric antigen receptors (CARs) are generated by fusing the variable regions of antibodies, costimulatory domain and TCR signaling domain. Accordingly, CAR-T can be activated by the targeted antigens (25). CAR-T is not the focus of this review, we refer readers to some elaborate reviews for more details (26, 27). In this part, we pay more attention to the ICIs targeting PD-1/PD-L1 signaling.

PD-1/PD-L1 signaling is a key factor of local immunosuppression in the tumor microenvironment. In inoperable advanced cancers, blockade of this pathway can suppress growth of cancers which are refractory to other therapies. The PD-1 blocking antibody nivolumab was approved in 2015 for treatment of advanced lung squamous cell carcinoma, marking the first clinical use of an anti-PD-1 therapy (28). There are three antibodies targeting PD-1 including pembrolizumab, nivolumab, and cemiplimab, and three blocking PD-L1 including atezolizumab, durvalumab, and avelumab (29). All these antibodies are approved by FDA for treatment of 17 different types of advanced unresectable cancers (29, 30). Different types of cancer, different patients in same cancer and even the same patient with different cancers show different degrees of response to ICIs targeting PD-1/PD-L1 signaling (31).

The antitumor effects of these drugs are very consistent, with primary response rates ranging from ~ 15% to ~65% for individual tumor types. Specifically, patients with renal cell carcinoma, NSCLC and melanoma have been evaluated for the response to atezolizumab and the overall response rate of patients with renal cell carcinoma is 30%, melanoma is 14% and NSCLC is 23% (32). In addition, a study on the efficacy of durvalumab in the treatment of locally advanced or metastatic urothelial carcinoma also showed that the overall response rate was 27.6% in patients with PD-L1 positive expression, compared to 5.1% in patients with PD-L1 negative expression (33). Various biological factors including lower tumor burdens, mutational burden, PD-L1 expression, and oncogenic virus integration are closely associated with a higher likelihood of response (34–39). However, several types of cancer, such as breast, prostate, pancreas and colon cancers, are particularly refractory to anti-PD-1/PD-L1 treatment (40).

Resistance is one of the critical challenges in ICIs (41, 42). More than half of the patients with cancer fail to respond to PD-1/PD-L1 inhibitors (43–46). There are two main manifestations of resistance including primary resistance, where cancer does not response to immunotherapy at all, and acquired resistance, where cancer responds to immunotherapy initially but relapses and progresses after a period of time. Both intrinsic factors and extrinsic factors contribute to immunotherapy resistance. Intrinsic factors include changed expression pattern of certain genes and pathways, expression of innate anti-PD-1 resistance signature, epigenetic modification of the DNA whereas extrinsic factors include immunosuppressive cells and factors within the tumor microenvironment (31, 47, 48). IFN-γ plays a key role in both primary and acquired resistance to checkpoint blockade therapy. It has both favorable and unfavorable effects on anticancer immune responses. Activated cancer-specific T cells recognize homologous antigens on cancer cells and produce IFN-γ, inducing an effective anticancer immune response. However, continued exposure to IFN-γ can increase the levels of PD-L1 in cancer cells, leading to immune escape (48–51). Recently, a series of studies reported that cancer cells stimulated by IFN-γ can release exoPD-L1, which possesses the ability to suppress anticancer immune responses and is associated with anti-PD-1 response (4, 5, 52, 53). More about exoPD-L1 will be discussed in the following content.

Although single-agent immune checkpoint inhibition was initially used, an increasing number of patients are treated with combination immune checkpoint blockade, in which the nonredundant mechanism of action of the individual agents often results in higher response rates. In addition, the immune checkpoint therapy has been combined with a variety of other treatments, including chemotherapy, radiotherapy and other immune therapies, such as vaccines, adoptive cell therapy, cytokines and so on, to maximize the clinical curative effect (53).

## 4 Action mechanisms and regulation of exoPD-L1

Given that PD-L1 has a typical structure of membrane-bound ligand protein, it is believed that PD-1/PD-L1 signaling can only function locally through intercellular contacts. However recent studies revealing that PD-1/PD-L1 signaling can be dispatched from the mothership as an expeditionary force and function systematically has change the stereotype. Activation of PD-L1 signaling inhibits cell cycle progression and proliferation of T cells (12), induces apoptosis of T cells (54) and induces regulatory T cells (Tregs) (55). As exoPD-L1 can be regarded as a potentiated PD-L1, exoPD-L1 should possess the same properties as membrane PD-L1. Some researches investigating exoPD-L1 directly reveal more details. In addition to inducing apoptosis (7, 56), exoPD-L1 inhibits TCA, including killing effect and cytokine production, in a dose-dependent manner by inhibiting ERK phosphorylation and NF-κB activation (5, 7, 52). It has been demonstrated that exoPD-L1 also suppresses TCA in draining lymph node *in vivo* (52). ExoPD-L1 exerts effect not only on T cells but also on dendritic cells (DCs). ExoPD-L1 from Lewis lung carcinoma or 4T1 breast cancer cell block the differentiation of DCs and induce apoptosis of DCs, and increasing the rates of Tregs consequently (57).

It has been reported that MHC I and II present on exosomes (58, 59). Given the interaction between MHC molecules and TCR, exoPD-L1 can take advantage of MHC to enhance its immunosuppressive function and exert a more robust immunosuppressive effects than the soluble form (60). A recent study revealed that intercellular adhesion molecule-1 (ICAM-1) is a prerequisite for exoPD-L1-mediated suppression of TCA. Furthermore, ICAM-1 and PD-L1 co-localize on exosomes and both can be upregulated by IFN-γ. Interacting with lymphocyte function-associated antigen-1 (LFA-1) on T cells, ICAM-1 potentiates the adhesion between exoPD-L1 and T cells (61).

In addition to secreting exoPD-L1 directly, cancer cells can induce other cells to secrete exoPD-L1. Specifically, HPV infection induces cervical cancer cells to secrete CXCL10 which binds to CXCR3 in the surrounding fibroblast cells to activate JAK-STAT pathway and produce exoPD-L1 (62). Anti-PD-1 treatment can suppress cancer growth in patients whose cancer cells do not express PD-L1, indicating that PD-L1 expression in non-cancer cells is also of great importance. As a matter of fact, exoPD-L1 can serve as a trafficking vehicle to deliver PD-L1 to different cell types including cancer cell, macrophage and DC in the tumor microenvironment (5). Furthermore, bone marrow-derived cells can also secrete exoPD-L1 to promote tumor metastasis by suppressing TCA (63). In glioblastoma, exoPD-L1 contributes to immunosuppression by inducing immunosuppressive nonclassical monocytes, rather than directly inhibiting TCA (64).

**Figure 2:**
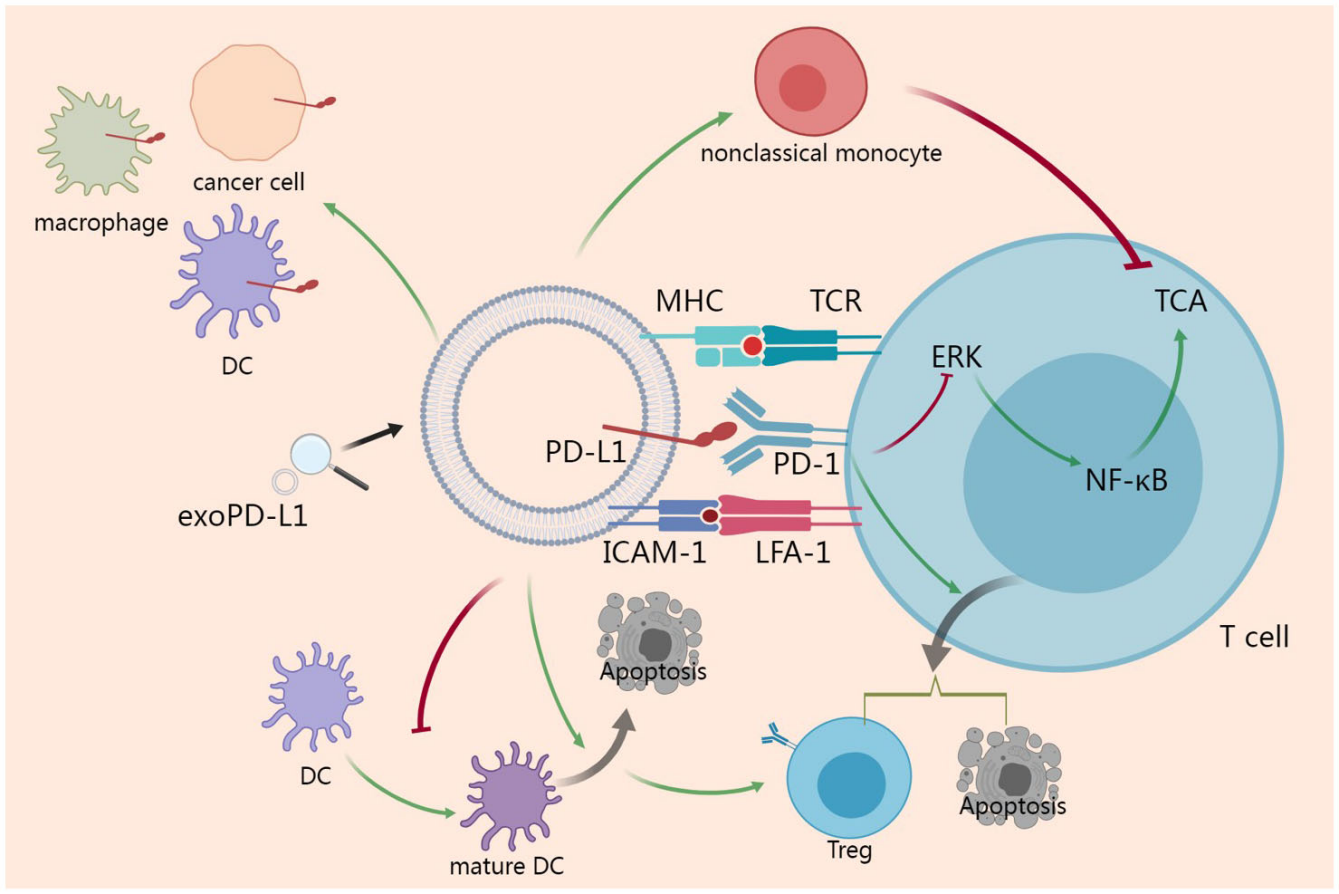
Action mechanisms of exoPD-L1. ExoPD-L1 take the advantages of MHC and ICAM-1 to potentiate the adhesion to T cell and inhibit TCA via ERK and NF-κB pathway. ExoPD-L1 can induce Treg and apoptosis of T cell. Additionally, exoPD-L1 blocks the maturation of DC and induces the apoptosis of DC, which promotes the induction of Treg. In addition to Treg, immunosuppressive nonclassical monocyte can also be induced by exoPD-L1. Finally, exoPD-L1 can deliver PD-L1 to different cell types including cancer cell, macrophage and DC in the tumor microenvironment.

Taken together, in tumor microenvironment, both cancer cells and non-cancer cells secrete exoPD-L1. Furthermore, exoPD-L1 serve as a trafficking vehicle to deliver PD-L1 to other cells. With MHC and ICAM-1 on surface, exoPD-L1 shows great adhesion to T cell and exert a more robust immunosuppressive effects than the soluble form. In addition to inhibiting TCA directly, exoPD-L1 can induce immunosuppressive nonclassical monocyte and Treg to suppress anti-cancer immune responses (**Figure 2**).

A series of factors, such as Ras-related protein GTPase Rab, Sytenin1, TSG101, apoptosis-linked gene 2-interacting protein X (ALIX), syndecan-1, the endosomal sorting complex required for transport complex (ESCRT) proteins, phospholipids, tetraspanins, ceramides, sphingomyelinases, and SNARE complex proteins, influence the origin and biogenesis of exosomes (65–68).

**Figure 3:**
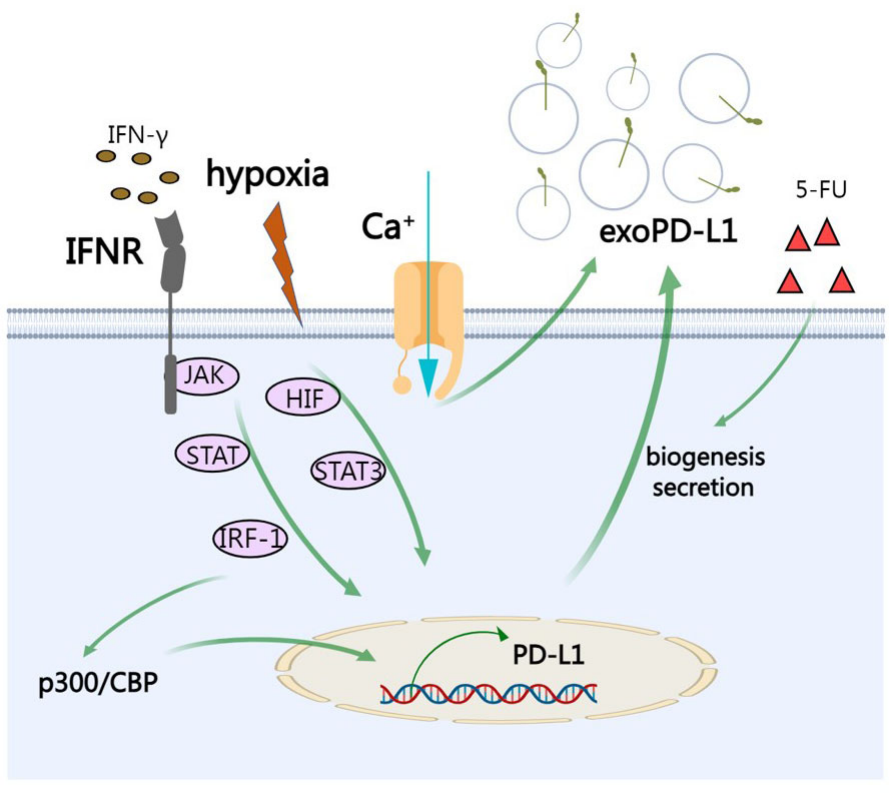
The regulation of exoPD-L1. IFN-γ activates JAK/STAT signaling and subsequent IRF-1, leading to production of exoPD-L1. Under hypoxic condition, hypoxia increases production of exoPD-L1 in a HIF-STAT3-dependent manner. Calcium signaling also promotes the secretion of exoPD-L1. Chemotherapeutic agent 5-FU can promote exoPD-L1 biogenesis and secretion. IRF-1 recruits p300/CBP to increase transcription of PD-L1.

External factors can also influence biogenesis of exosomes including cell type, cell confluency, serum conditions, cytokines, growth factors, the sites of exosomes, protein sorting, physico-chemical aspects, etc. (69) PD-L1 on cell surface can be upregulated by IFN-γ and it has been reported that exoPD-L1 from melanoma cells can be also augmented upon IFN-γ treatment (4, 52). Specifically, after the engagement on interferon-gamma receptor, IFN-γ activates JAK/STAT signaling and subsequent IRF-1, leading to production of exoPD-L1 (70). Calcium signaling is involved in exosome biogenesis and secretion. Accordingly, targeting calcium signaling can inhibit the expression of exoPD-L1 (71). Under hypoxic condition, hypoxia increases production of exoPD-L1 in a hypoxia-inducible factors (HIF)-STAT3-dependent manner (71). Therapeutic interventions such as chemotherapeutic agent 5-fluorouracil (5-FU) can promote exoPD-L1 biogenesis and secretion in advanced GC (56). IRF-1 can recruit p300/CBP to increase transcription of PD-L1 and abrogating this process by a p300/CBP inhibitor intensely increased the efficacy of PD-L1 blockade treatment in prostate cancer (72) (**Figure 3**).

## 5 Functions of exoPD‐L1 as biomarkers

A great number of studies have regarded exoPD-L1 as a tumor diagnostic and prognostic marker (73–75). ExoPD-L1 is closely associated with disease progression, such as bigger tumor size, metastasis, lower overall survival (OS) and more advanced TNM stage in melanoma (4, 52, 76), NSCLC (77–79), head and neck squamous cell carcinomas (HNSCC) (80–82), GC (6), osteosarcoma (83) and diffuse large B-cell lymphoma (DLBCL) (84). In pancreatic cancer, serum samples from 55 patients with pancreatic ductal adenocarcinoma (PDAC) have been analyzed for levels of exoPD-L1, which have no difference from those of patients with chronic pancreatitis and benign serous cyst adenoma of the pancreas (74). However, exoPD-L1-positive PDAC patients have shorter postoperative survival time, indicating that exoPD-L1 can be a negative prognostic factor rather than a diagnostic biomarker for pancreatic cancer (74). In GC, high exoPD-L1 group show lower OS and exoPD-L1 is an independent prognostic factor (6). In addition to the basal expression of exoPD-L1, variations of exoPD-L1 (ΔExoPD-L1) after cures is also associated with prognosis. ΔExoPD-L1> 100pg/mL is regarded as predictive marker for disease progression in melanoma patients (73). Similar work showed that HNSCC patients with recurrence during treatment had increases in exoPD-L1 from baseline (85). The advantage of exoPD-L1 as diagnostic and prognostic markers lies on its convenience and noninvasiveness, as it is easier to obtain liquid biopsy than reaching tumor niche. Furthermore, compared with PD‐L1 expression in tumor biopsies, circulating exoPD-L1 may be a more reliable marker (73).

As a great proportion of cancer patients fail to respond to anti-PD-1/PD-L1 therapy, it is urgent to find a predictive marker for anti-PD-1/PD-L1 therapy. Recently, exoPD-L1 has been regarded as a reliable predictor for immunotherapy and used as a marker discriminating responders from non‐responders. It is intuitive that a high level of exoPD-L1 reflects suppressed immune responses against cancer. However, responders of anti‐PD‐1 treatment show an increase of exoPD-L1 after treatment and there is no significant difference of exoPD-L1 level in non‐responders after treatment (4). Level of exoPD-L1 before treatment reflects severity of immune dysfunction. In patients failing to respond to the anti-PD-1 treatment, level of exoPD-L1 is significantly higher than that of responders and the functions of T cells are severely damaged to a state that they cannot be reinvigorated by anti-PD-1 treatment (4). Similar results have also been identified in oral cancer (86). During anti-PD-1 treatment, an increase in the level of exoPD-L1, induced by IFN-γ, reflects T cell reinvigoration and a successful anticancer immunity. Given that the PD-1/PD-L1 signaling is blocked during anti-PD-1 therapy, the increased exoPD-L1 is unable to suppress TCA (4). On the whole, responders to anti-PD-1 treatment have low levels of exoPD-L1 before treatment and show significant increase of exoPD-L1 during treatment. Accordingly, a fold change in circulating exoPD-L1 greater than 2.43 during immunotherapy is associated with better therapeutic effects (4). However, increase of exoPD-L1 during treatment is not always associated with good results (73). The contradictions and controversies in this topic may lie in differences of monitor time point of exoPD-L1 (87). Fold change of exoPD-L1 at early time (at week 3–6) reflects T cell reinvigoration and is reasonably higher in responders (4). However, after several months of treatment (median interval is 4.5 months), responders show obvious tumor regression and exoPD-L1 decrease accordingly, while non-responders show tumor progression which increases the levels of exoPD-L1 (73). Taken together, both fold change of exoPD-L1 and monitoring time should be taken into account to distinguish responders from non-responders.

In addition to exoPD-L1, exosomal PD-1 and exosomes containing PD-L1 mRNA are also associated with response to anti-PD-1/PD-L1 treatment (88, 89). Furthermore, exoPD-L1 and CD28 can be combined to be a more reliable predictive biomarker for clinical responses to anti-PD-1 treatment (90). Despite that most of the researches rely on exoPD-L1 from plasm, it should note that other bodily fluids, such as saliva, urine, or cerebrospinal fluid, may also serve as viable sources for exoPD-L1. For example, exoPD-L1 in urine samples from patients with urothelial cancer is also associated with responses of anti-PD-L1 therapy (91).

## 6 New technologies for analysis of exoPD-L1

There are some conventional detection methods for exoPD-L1. For example, electron microscopy and immuno-electron microscopy are qualitative detection methods to demonstrate the existence of exoPD-L1 (87). The percentage of exoPD-L1 can be measured by nanoscale flow cytometry or traditional flow cytometry with the help of magnetic beads or latex beads. The relative quantification of PD-L1 level can be reflected by the relative fluorescence intensity. Western blotting is used to evaluate the level of total PD-L1 protein in exosome. Both flow cytometry and western blotting are semiquantitative detection methods. Enzyme-linked immunosorbent assay (ELISA) is used for absolute quantitative detection. However, Western blotting and ELISA have detection limit and are not suitable for detecting low abundance of exoPD-L1 at the early stage of cancer. Furthermore, these conventional detection methods are both sample-consuming and time-consuming (87). Accordingly, researchers have been making the effort to develop novel methods to detect exoPD-L1 (87, 92) (**Table 1**).

**Table 1:** New technologies for analysis of exoPD-L1.

Platform	Sensing mechanism	Advantages	Sample source	Reference
SERS	Fe3O4@TiO2 nanoparticles are used to enrich exoPD-L1 and Au@Ag@MBA SERS tags are used for quantification.	1.The whole process can be finished within 40 min. 2.ExoPD-L1 can be quantified by using a 4 μL clinical serum sample. 3.NSCLC patients can be distinguished from the healthy controls easily.	NSCLC	(79)
Electrochemistry	ExoPD-L1 is captured by anti-CD63 magnetic beads and a typical DNA amplification reaction is conducted, leading to disassembly of PVP@HRP@ZIF-8 and arousing of amplified electrochemical responses.	ExoPD-L1 can be found in the undiluted serum samples from patients with breast cancer, particularly for metastatic breast cancer	TNBC	(93)
Thermophoresis	The method combines a newly evolved aptamer that binds to PD-L1, and homogeneous thermophoresis with a rapid binding kinetic.	Sensitive, quick and easy to operate	adenocarcinoma	(94)
LSPR	Au@Ag NBPs capture exoPD-L1 and anti-hPD-L1–AuNR is added to the system to emit signals.	This method allows rapid, highly sensitive exosome detection and accurate identification of PD-L1 exosome subtypes in a single assay.	_	(95)
DCNP	DNA molecular machine-based dual-recognition probes are fitted onto gold nanoparticle surface to initiate signal-amplified synchronous response	This method can simultaneously detect exoPD-L1 and exosomal miRNA-21.	breast cancer	(96)
SPR	An intensity-modulated, compact SPR biosensor is developed.	This method shows higher detection sensitivity and similar sensing accuracy when compared with ELISA.	NSCLC	(78)
ExoHCR	ExoPD-L1 is captured by αCD63-conjugated magnetic beads and incubated with a conjugate of PD-L1 antibody to initiate HCR.	Non-invasive, sensitive, and fast	melanoma colorectal cancer	(97)
TRACER	Two aptamers targeting tumor biomarker EpCAM and PD-L1 are utilized.	Accurate quantification of tumor-derived exoPD-L1 with high sensitivity and selectivity	A375 cell	(98)
TRACER	An aptamer- and lectin-induced proximity ligation assay combined with quantitative real-time polymerase chain reaction is developed.	This method enables glycosylated exoPD-L1 quantitation with high sensitivity and selectivity in a wash-free manner.	A375 cell	(99)
FRET	The glycosylation of exoPD-L1 is imaged *in situ* using intramolecular fluorescence resonance energy transfer.	This method enables *in situ* visualization of glycosylated exoPD-L1.	A375 cell	(100)

There is a sandwich exosome immunoassay which permits quantification and separation of exoPD-L1 according to the levels of PD-L1 expression in a single assay (95). Specifically, gold–silver core–shell nanobipyramids (Au@Ag NBPs) serve as the primary capture layer to bind exoPD-L1 and release the first signal through an LSPR dark-field imaging technique. Later, anti-hPD-L1 antibody-functionalized AuNR (anti-hPD-L1–AuNR) is added to the system to emit a secondary signal. This method generates a two-step LSPR signal pattern and levels of PD-L1 on exosome can be differentiated according to the secondary LSPR signal intensity (95).

Cao et al. proposed an electrochemical biosensing method for quantification of exoPD-L1 by using DNA amplification-responsive metal-organic frameworks, PVP@HRP@ZIF-8 (93). The detection limit reaches 334 particles/mL by the biosensing method (93). Specifically, PVP@HRP@ZIF-8 is a pH-responsive compound which is prepared by encapsulation of HRP into ZIF-8 and coated by PVP. ExoPD-L1 is captured by anti-CD63 magnetic beads and capture probe is linked to antibody targeting PD-L1 on exosome. Capture probes of this sandwich-like construction function as primers to initiate hyperbranched rolling circle amplification, a typical DNA amplification reaction. As the reaction progress, environmental pH decrease, leading to the release of enzymes and arousing amplified electrochemical responses. Accordingly, exoPD-L1 can be identified (93).

A method which can simultaneously detect exoPD-L1 and exosomal miRNA-21 has been developed (96). Despite their heterogeneity, low abundance and spatial segregation, a dual-cycling nanoprobe (DCNP) has been designed to enable single-step simultaneous quantitation. Specifically, DNA molecular machine-based dual-recognition probes are fitted onto gold nanoparticle surface to form DCNP and this can initiate signal-amplified synchronous response by exosomal miR-21 and exoPD-L1 within 20 mins (96).

Surface plasmon resonance (SPR) is a real-time optical detection method with high sensitivity, label-free and timesaving property. Liu et al. reported a method using a compact SPR biosensor to detect exoPD-L1 in which the biochip surface was improved by the mixture of PEG200 and biotin-PEG1000, Neutravidin, and biotinylated antibodies to maximize the capture efficiency of exoPD-L1 (78). Additionally, this method was compared with ELISA and shown higher detection sensitivity and similar sensing accuracy (78).

Huang et al. developed a method of exoPD-L1 quantitation with a homogeneous, low-volume, efficient, and sensitive property (94). The method combined aptamer and thermophoresis. The aptamer is a short single strand DNA with higher recognition efficiency than antibody for exoPD-L1 whereas thermophoresis is the reaction system with high detection sensitivity, efficient reaction rate, separation-free and homogeneous nature. Due to the binding-induced change in Soret coefficient, the thermophoresis of exosome-aptamer complex is different from the PD-L1 aptamer. In addition, thermophoretic depletion of unbound PD-L1 aptamer is faster than that of exosome-aptamer complex, and, accordingly, the complex tends to retain whereas free aptamer is prone to be depleted. Under excitation, fluorescence intensity of dye-labeled complex is monitored and fluorescence intensity is correlated with the concentration of exoPD-L1 (94). This method showed great accuracy in cancer diagnosis (AUC: 0.999) and is more sensitive than ELISA-based method (94).

Hu et al. reported a non-invasive, sensitive, and fast assay to quantify levels of exoPD-L1, which was referred to as exosome-hybridization chain reaction (ExoHCR). Specifically, αCD63-conjugated magnetic beads are utilized to isolate exoPD-L1 which is then incubated with a conjugate of PD-L1 antibody with an HCR trigger DNA (T). A pair of metastable fluorophore-labeled hairpin DNA (H1 and H2) was added subsequently, initiating HCR *in situ* on bead-conjugated exosome surfaces. Finally, ExoHCR amplified the fluorescence signal intensities by 3-7 times and facilitated detection of exoPD-L1 greatly (97).

Pang et al. recently developed a method integrating capture and analysis of exoPD-L1 directly from serum. Firstly, Fe_3_O_4_@TiO_2_ nanoaparticles are synthesized to enrich exoPD-L1 by the binding of TiO_2_ shell and hydrophilic phosphate head of phospholipids on exoPD-L1. ExoPD-L1 can be enriched within 5 mins and capture efficiency is up to 96.5%. Secondly, after enrichment, anti-PD-L1 antibody modified Au@Ag@MBA SERS tags are used to label the exoPD-L1 for Raman signal detection. The whole detection process finish within 40 mins. Furthermore, with only 4 μL clinical serum sample, this method can be used for exoPD-L1 quantification to distinguish NSCLC patients from the healthy controls (79).

The accurate quantitation of tumor-derived exoPD-L1 was hindered by the exoPD-L1 from normal cells which are also abundant in body fluids. An excellent method named dual-target-specific aptamer recognition activated *in situ* connection on exosome membrane combined with droplet digital PCR (ddPCR) (TRACER) has been developed by Lin et al. to achieve accurate quantification of tumor-derived exoPD-L1 with high sensitivity and selectivity (98). Specifically, two aptamers, which target tumor biomarker EpCAM and PD-L1 respectively, are utilized. Tumor-derived exoPD-L1 can simultaneously bind to these two aptamers which will be close to each other because of the fluidity of the exosome membrane. Later, the extended ends of two aptamers are ligated via PLA and droplet digital PCR (ddPCR) is performed in site, quantifying tumor-derived exoPD-L1 absolutely (98). With a similar method, glycosylated exoPD-L1 can also be quantified (99). Furthermore, glycosylated exoPD-L1 can be visualized *in situ* by imaging-based method (100) (**Table 1**).

## 7 Targeting exoPD-L1 to restore T cell response and enhance immunotherapy

Some pioneering researches have revealed the importance of exoPD-L1 in anticancer immunity and anti-PD-L1 therapy (4, 5, 52). Removal of exoPD-L1 can be achieved via genetic deletion of some key genes, including Rab27A, aSNase2, Hrs, ALIX, histone lysine-specific demethylase 1 (LSD1) and PD-L1 (4, 5, 52, 101, 102). Additionally, pharmacological approaches such as exosome secretion inhibitor GW4869 can also inhibit exoPD-L1 (5). In a mouse model of prostate cancer which is resistant to anti-PD-L1 antibody, prostate cancer cells fail to grow in mice when Rab27A or aSNase2 was deleted (52). In addition to cancers resistant to anti-PD-L1 therapy, cancer growth of MC38 mice, a colorectal cancer model which shows a partial response to anti-PD-L1 therapy, is also suppressed by Rab27A knockout (52). Interestingly, in mouse model which is resistant to anti-PD-L1 antibody, there is no difference between deletion of exoPD-L1 and deletion of the whole PD-L1. However, in mouse model which shows a partial response to anti-PD-L1 therapy, the whole PD-L1 loss has a greater effect than exoPD-L1 loss, indicating that exoPD-L1 plays an important, but partial role, in this model (52). In addition to direct inhibition of cancer growth, blocking exoPD-L1 also induces an abscopal effect, a phenomenon that treatment of local cancer gives rise to regression of a distant cancer (52). Specifically, In the mouse prostate cancer model, mutant cancer cells lacking exoPD-L1 and wild type (WT) cancer cells are injected into the opposite sides of mice. Surprisingly, the growth of WT cancer cells can be greatly reduced (52). Furthermore, blocking exoPD-L1 can induce anticancer memory response against secondary challenges of cancer cells (52). Specifically, the mice injected with cells lacking exoPD-L1 can survived more than 90 days, and the surviving mice are reinjected with WT cells on the opposite flank. Surprisingly, WT cancer cells fail to grow in mice that are pre-challenged by mutant cells (52). Similarly, inhibiting exoPD-L1 secretion in 4T1 mouse mammary cancer cells also significantly inhibit 4T1 cancer growth *in vivo* (5). Interestingly, compared with anti-PD-L1 antibody, suppressive effect of inhibiting exoPD-L1 secretion is more profound, underling the importance of exoPD-L1 in suppressing anticancer immunity (5). Furthermore, combination of inhibiting exoPD-L1 secretion with PD-L1 antibody can significantly augment anti-PD-L1 therapeutic efficacy (5).

Taken together, both exoPD-L1 and membrane PD-L1 are involved in immunosuppression. Immunotherapy targeting PD-L1 mainly functions by blocking membrane PD-L1 and cancer cells secreting high levels of exoPD-L1 are prone to resist anti-PD-L1 therapy. Accordingly, the distribution of PD-L1 between exosome and cell surface is a determining factor of immunotherapy. Blocking exoPD-L1 secretion of cancer cells can suppress cancer growth locally, induce an abscopal effect and an anticancer memory. More importantly, inhibiting exoPD-L1 secretion can augment anti-PD-L1 therapeutic efficacy and targeting exoPD-L1 may overcome the resistance to anti-PD-L1 therapy.

Biogenesis of exoPD-L1 is a complicated process and there are different ways to inhibit exoPD-L1. When genetic approaches are adopted, some researches have come to opposite conclusion. For example, Knockdown of ALIX, an ESCRT accessory protein, can decrease exoPD-L1 production in breast cancer, but promotes the cancer growth by elevating membrane PD-L1 (102). Additionally, given that genetic approaches can hardly be used in human beings, pharmacological approaches have gained a lot of attention. Anticancer effect of atorvastatin has been identified in mouse model and when combined with anti-PD-L1 antibody, it can improve the effect of anti-PD-L1 therapy (103). In terms of mechanisms, atorvastatin inhibits exoPD-L1 by regulating the Rab proteins and influencing mitogen-activated protein kinases (MAPK) signaling pathway (103). Macitentan, an FDA-approved oral drug, can also inhibit exoPD-L1 by targeting the endothelin receptor A in breast cancer cells and in xenograft mouse model (104). Sulfisoxazole, a sulfonamide antibacterial, exerts robust anticancer effects by inhibiting exoPD-L1 (105, 106). Temsirolimus, a targeted anti-cancer drug, inhibiting exoPD-L1 by activating autophagy (107). Recently, Sun et al. identified an inhibitor of exoPD-L1 as a sensitizer of to promote immunotherapy in GC (108). They have explored 2791 compounds to identify the most effective inhibitor of exoPD-L1 which was referred to as EP16 (108). The function of EP16 was confirmed both *in vitro* and *in vivo*. Both EP16 and anti-mouse PD-1, can inhibit the growth of the cancer, with a tumor growth inhibitory rate (TGI) of 32% and 51% respectively. Combination of EP16 with anti-mouse PD-1 promotes the TGI up to 69% (108).

Recently, some new methods inhibiting exoPD-L1 have been developed. Ferroptosis can generate oxidative hydroxyl radicals and this oxidative cell death was reported to enhance anticancer immunity (109). Wang et al. have developed nanoparticles which are composed of GW4869 and ferroptosis inducer to suppress B16F10 melanoma cells and elevate response to anti-PD-L1 treatment (110). ExoPD-L1 and many types of virus particle have cell-derived membrane envelopes which inspired researchers that antiviral curvature-sensing peptides may also disrupt membrane-enveloped exoPD-L1 (111). An engineered antiviral peptide has been repurposed to disrupt exoPD-L1 and its effect is associated with the pH of cancer microenvironment (111). The advantages of RNA interference comprise excellent specificity, high sensitivity, fast response, and strong gene silencing efficiency. However, efficiency of siRNA is determined by the delivery vector. Zhang Sun et al. has developed biomimetic exosomal vesicles loaded with siRNA to inhibit the secretion of exoPD-L1 recently (112). Specifically, they combined exosome membrane, apoA1 and phospholipid into biomimetic exosome vesicles (apoA1-bExo) and incubated apoA1-bExo with cholesterol modified siRNA to form apoA1-bExo containing siRNA (apoA1-bExo/siRNA). Accordingly, apoA1-bExo/siRNA can take advantage of endosome-Golgi-ER pathway and selective uptake pathways to enter cancer cells and reduce secretion of exoPD-L1 (112). Its effects to block exoPD-L1 has also been confirmed in an immune system-cancer dual humanized mice (112).

On the whole, both exoPD-L1 and membrane PD-L1 are the focus of future therapeutic strategies and blocking two of them at the same time will be a promising strategy to cure cancers in clinical practice. Additionally, exosomes are involved in a variety of physiological processes and inhibiting exosomes may interfere with functions of exosomes from healthy cells. Accordingly, immunotherapies targeting exoPD-L1 should be conducted with caution to prevent adverse effects. Although therapeutic agents for exoPD-L1 is still in infancy, all these preclinical studies provide a new direction for cancer treatment in the future.

## 8 Functions of exoPD-L1 beyond cancer: the beneficial ones

Uncontrolled inflammation after injury can lead to chronic wounds and effective wound repair requires appropriate inflammatory responses. Su et al. investigated the role of exoPD-L1 in wound healing (113). They obtained high concentration of exoPD-L1 from genetically engineered cells overexpressing PD-L1 or IFN-γ stimulated cells, and revealed that exoPD-L1 can promote the migration of epidermal cells and dermal fibroblasts (113). Furthermore, they embedded exoPD-L1 into thermoresponsive hydrogel, which can be gelatinized at body temperature to release exoPD-L1 to the surroundings in a sustained manner. ExoPD-L1 can fasten wound contraction and reepithelialization was also confirmed in a mouse skin excisional wound model (113).

Allogeneic hematopoietic cell transplantation (HCT) is a predominant treatment for hematological malignancies. However, HCT may lead to acute graft-versus-host disease (aGvHD), a serious complication in which donor T cells recognize and attack the recipient’s non-malignant tissues. Mesenchymal stem cells (MSCs) possess immunomodulatory properties that are beneficial for the treatment of aGvHD. Li et al. investigated the role of exoPD-L1 from Wharton’s Jelly-derived MSCs (WJMSCs) in aGvHD. They revealed a rapid increase of exoPD-L1 in patients with aGvHD after the infusion of WJMSCs and exoPD-L1 can inhibit TCA (114).

## 9 Conclusion

PD-1 is one of the most famous co-inhibitory receptors on the surface of activated T cells. It should keep in mind that PD-1/PD-L1 signaling comprises the ‘reverse signaling’ which is down from PD-L1. However, precise signal transduction pathway of ‘reverse signaling’ is not well understood. Over the last decades, immunotherapy for cancers, especially ICIs targeting PD-1/PD-L1 signaling, have achieved tremendous success. However, not all cancers response to immune therapies. A series of studies has reported that PD-L1 can be secreted in form of exosomes at the extracellular level, which is involved in resistance to anti-PD-1 treatment. A range of factors, including IFN-γ, hypoxia, calcium signaling and chemotherapeutic agent 5-FU can regulate the formation of exoPD-L1. The molecular mechanisms underlying the exoPD-L1 have been disclosed. ExoPD-L1 take the advantages of MHC and ICAM-1 to potentiate the adhesion to T cell. ExoPD-L1 can induce immunosuppressive Treg and nonclassical monocyte. Finally, exoPD-L1 can deliver PD-L1 to different cell types in the tumor microenvironment. As a matter of fact, the research of exoPD-L1 is still in the early stage and the knowledge of exoPD-L1 is limited.

Emerging evidence has shown that exoPD-L1 can be biomarkers for diagnosis and prognosis of cancer and biomarkers for predicting responses of immune therapies. However, there are some contradictions and controversies in this topic and this need further investigation in the future. As circulating exoPD-L1 can be a more reliable marker than PD‐L1 in tumor biopsies, more and more methods analyzing exoPD-L1 have been developed. Currently, these methods are still in the laboratory and have not been extended to clinical practice, which should be addressed in the future.

Therapeutic methods targeting exoPD-L1, especially pharmacological ones, to restore T cell response and enhance immunotherapy have gained a lot of attention. Oral drugs for the treatment of other diseases, newly identified compounds, novelly designed nanoparticles, etc. are used to inhibit exoPD-L1. Although therapeutic agents for exoPD-L1 is still in infancy, all these preclinical studies pave the way for cancer treatment in the future.

As a matter of fact, exoPD-L1 is not always a bad character. Functions of exoPD-L1 beyond cancer have been brought into focus recently. Essence of exoPD-L1 as an immunosuppressant provides a theoretical and experimental basis for treatments of disorders such as organ transplantation rejection, autoimmune diseases, chronic infection, chronic inflammation, etc.

## Author contributions

ML: Conceptualization, Writing – original draft. YY: Conceptualization, Writing – review & editing.

## Glossary

**Table:**

PD-1	programmed cell death protein 1
TCA	T cell activation
exoPD-L1	exosomal PD-L1
IFN-γ	interferon-γ
GC	gastric cancer
NSCLC	non-small cell lung cancer
ICI	immune checkpoint inhibitor
Ig	immunoglobulin
MHC I	major histocompatibility complex class I
APC	antigen-presenting cell
TCR	T cell receptor
ITIM	immunoreceptor tyrosine-based inhibitory motif
ITSM	immunoreceptors tyrosine-based switch motif
SHP-1	Src homology 2 domain-containing protein tyrosine phosphatase 1
PI3K	phosphoinositide 3-kinase
ERK	extracellular-signal-regulated kinase
ICB	immune checkpoint blockade
CAR-T	chimeric antigen receptor T-cell
CAR	chimeric antigen receptor
HIF	hypoxia-inducible factors
5-FU	5-fluorouracil
Treg	regulatory T cell
DC	dendritic cell
ICAM-1	intercellular adhesion molecule-1
LFA-1	lymphocyte function-associated antigen-1
ALIX	apoptosis-linked gene 2-interacting protein X
ESCRT	the endosomal sorting complex required for transport complex
OS	overall survival
HNSCC	head and neck squamous cell carcinomas
DLBCL	diffuse large B-cell lymphoma
PDAC	pancreatic ductal adenocarcinoma
ELISA	enzyme-linked immunosorbent assay
SPR	surface plasmon resonance
TNBC	triple negative breast cancer
LSD1	histone lysine-specific demethylase 1
WT	wild type
MAPK	mitogen-activated protein kinases
HCT	hematopoietic cell transplantation
aGvHD	acute graft-versus-host disease
MSC	mesenchymal stem cell
WJMSC	Wharton’s Jelly-derived MSC.
